# Efficient and High-Quality RNA Isolation from Metabolite-Rich Tissues of *Stevia rebaudiana*, an Important Commercial Crop

**DOI:** 10.21315/tlsr2019.30.1.9

**Published:** 2019-01-31

**Authors:** Tan Yoeng Leh, Christina Seok Yien Yong, Rosimah Nulit, Janna Ong Abdullah

**Affiliations:** 1 Department of Biology, Faculty of Science, Universiti Putra Malaysia, 43400 UPM Serdang, Selangor, Malaysia; 2 Department of Cell and Molecular Biology, Faculty of Biotechnology and Biomolecular Sciences, Universiti Putra Malaysia, 43400 UPM Serdang, Selangor, Malaysia

**Keywords:** Conventional Method, Cetyltrimethylammonium Bromide (CTAB), Lithium Chloride, RNA Extraction, RNA Integrity, Kaedah Konvensional, Setiltrimetilamonium Bromida (CTAB), Litium Klorida, Pengekstrakan RNA, Integriti RNA

## Abstract

*Stevia rebaudiana*, a perennial herb native to northeastern Paraguay, has gained immense attention globally over the recent decades due to the natural sweetness of its leaves. Like in most plants, this particular species contains high amount of secondary metabolites, thus rendering the isolation of high quality and quantity RNA extract for molecular applications rather challenging. An effective, high-yield and high-quality RNA isolation protocol for this economically important plant species was devised here based on the cetyltrimethylammonium bromide (CTAB) extraction method, with an additional genomic DNA (gDNA) removal step. DNA and other contaminants that may affect downstream applications were effectively removed. Our results exhibited that RNA samples isolated from the leaves and stems of *Stevia rebaudiana* using this improvised method are high in integrity and quality with RNA integrity number (RIN) of more than 8 and low in contaminants.

## Abstract (ms)

*Stevia rebaudiana*, satu tumbuhan herba saka yang berasal dari timur laut Paraguay, telah mendapat perhatian yang banyak untuk beberapa dekad kebelakangan ini kerana kemanisan semula jadi daun-daunnya. Seperti kebanyakan tumbuhan, spesies ini mengandungi kandungan metabolit sekunder yang tinggi, dengan itu menyebabkan pengasingan RNA yang berkualiti dan berkuantiti tinggi untuk aplikasi-aplikasi hiliran menjadi agak mencabar. Sebuah protokol pengasingan RNA yang efektif, dapat memberikan hasil yang banyak dan berkualiti tinggi telah dirumuskan berdasarkan kaedah pengekstrakan setiltrimetilamonium bromida (CTAB), dengan penambahan langkah pembuangan DNA genom (gDNA). DNA dan bahan kontaminasi lain yang berkemungkinan mempengaruhi aplikasi hiliran juga disingkirkan secara efektif. Hasil kajian ini menunjukkan bahawa sampel-sampel RNA yang diasingkan daripada daun-daun dan batang-batang *Stevia rebaudiana* menggunakan kaedah yang direka ubah ini adalah dalam kualiti dan integriti yang tinggi dengan nombor integriti RNA (RIN) yang melebihi 8 dan tahap pencemaran yang rendah.

## INTRODUCTION

*Stevia rebaudiana* (Bertoni) is an herbaceous perennial plant of the Asteraceae family that is mostly grown in tropical and subtropical climatic regions (Yadav *et al*. 2011). This plant is native to the Amambay region in the northeastern part of Paraguay and also occurs in the neighbouring parts of Argentina and Brazil (Lemus-Mondaca *et al*. 2012). It is commercially well-known for its natural sweetness, and many research are being carried out investigating the nutritional and pharmacological aspects of this plant (Yadav & Guleria 2012). The plant is sweet in nature due to the presence of diterpene glycosides that are mainly found in its leaves. Leaf extract from *Stevia rebaudiana* is excellent as natural sweetener to replace sugar because of their non-caloric property, high sweetness level and even as potential relieving agents against various diseases (Yadav & Guleria 2012). The exploitation of these sweetening compounds has increased considerably due to health concerns related to sucrose consumption, such as dental caries, diabetes and obesity (Gantait *et al*. 2015). Nowadays, *Stevia rebaudiana* is getting more attention as a healthier alternative sweetener with zero calories and non-toxic in nature to control obesity, diabetes and high blood pressure over synthetic sweeteners such as acesulfame-K, aspartame, neotame, saccharin and sucralose that have been shown to be carcinogenic and are associated with many diseases (González *et al*. 2014; Lemus-Mondaca *et al*. 2015). Although considerable amount of research has been carried out on this plant, studies investigating genetic background such as the molecular mechanisms controlling the biosynthesis of the diterpenoids and other secondary metabolites that give *Stevia rebaudiana* its commercial value are still limited. This may partly due to the difficulties in isolating high quality nucleic acid samples from this plant.

Ribonucleic acid (RNA) extraction from plant tissue is always challenging. Plants contain polysaccharides and secondary metabolites such as polyphenolic compounds that could interfere with the quality and quantity of the RNA extract by co-precipitating with or binding to RNA and resulting in poor yields (Umesh *et al*. 2017). Like most plants, *Stevia rebaudiana* contains high amount of secondary metabolites including alkaloids, flavonoids, glycosides, saponins and tannins (Wölwer-Rieck 2012; Siddique *et al*. 2014). The presence of these compounds can greatly affect the successfulness of RNA extraction, let alone high-quality RNA extract. Furthermore, failing to remove these contaminants in the RNA extract may also limit and inhibit downstream molecular applications. It is desirable and essential to have high quality RNA sample for sensitive applications such as RNA sequencing, isolation of full length coding gene and functional genomic studies.

However, the successfulness of extracting high quality RNA from plant is strongly dependent on nature of the tissue, condition of the sample and species. Different plant samples or tissues, or species require different approaches for RNA extraction, it is very unlikely to have a single standard protocol that works on different tissues and plants. Several studies had been reported on the isolation of RNA from this plant using conventional methods based on acid guanidinium thiocyanate-phenol-chloroform (Michalec-Warzecha *et al*. 2016) and phenol/SDS based IHBT (Modi *et al*. 2014); as well as commercial kits (J Chen *et al*. 2014; Kim *et al*. 2015) with various degree of RNA purity and quantity. Here, we developed an efficient and high-quality yield RNA extraction protocol for this economically important species by modifying a previously reported CTAB method (Chang *et al*. 2016). To our best knowledge, this is the first CTAB-based RNA extraction protocol developed for *Stevia rebaudiana*. Analyses of the RNA extracts showed high quality and yield of RNA obtained and we recommend this improved CTAB protocol to others who require high quality and quantity RNA in their work on *Stevia rebaudiana*.

## MATERIALS AND METHODS

### Plant Materials

The first three nodes of young leaf and stem tissues at the budding stage of *Stevia rebaudiana* were collected, immediately frozen in liquid nitrogen for transportation, and later stored in −80°C freezer until RNA was ready to be extracted.

### Solutions and Reagents

All glass apparatus, mortar and pestle, and plastic wares were pre-treated with 0.1% DEPC (diethylpyrocarbonate) overnight and then autoclaved at 121°C for 45 min before being used for RNA isolation. All solutions were prepared with distilled water pre-treated with 0.1% DEPC. The extraction buffer used for this study contained: 2% CTAB (cetyltrimethylammonium bromide), 100 mM Tris-HCl (pH 8), 2M NaCl, and 25 mM EDTA (ethylenediaminetetraacetic acid). The other solutions and reagents used include: PVP (polyvinylpyrrolidone, average MW 40000), β-mercaptoethanol, chloroform: isoamyl alcohol (24:1), 8M lithium chloride (LiCl_2_), 70% and 100% ethanol (ice-cold), RQ1 RNAse-Free DNAse (Promega, Wisconsin, USA), and DEPC-treated and -autoclaved distilled water. All chemicals used were of molecular grades.

### RNA Extraction Procedure

Prior to pre-warming at 65°C in dry bath, 0.014 g of PVP and 14 μL of β-mercaptoethanol were added to 686 μL of extraction buffer. In the meantime, 0.1 g of frozen leaf (or stem) samples were ground into fine powder using pre-chilled mortar and pestle in the presence of liquid nitrogen. The fine powder was then transferred into a 1.5 mL microcentrifuge tube and 700 μL of pre-warmed CTAB extraction buffer containing PVP (2% w/v) and β-mercaptoethanol (2% v/v) were added immediately. The sample was vortexed twice, each time for 10 s, and immediately incubated at 65°C for 20 min with gentle mixing by inverting the 1.5 mL tube every 5 min. An equal volume of chloroform: isoamyl alcohol (24:1) was added into the 1.5 mL tube containing the sample, and vortexed until the mixture was homogenous. The mixture was then centrifuged at 16500 ×*g* for 15 min at 4°C. After centrifugation, the supernatant was then transferred to a new 1.5 mL tube and re-extraction was carried out by adding an equal volume of chloroform: isoamyl alcohol (24:1). The 1.5 mL tube was shaken a few times for mixing and centrifuged at 16500 ×*g* for 10 min at 4°C. Then, the supernatant was transferred into a new 1.5 mL tube and 1/3 volume of 8M LiCl_2_ was added, mixed well and the sample was precipitated at −20°C overnight.

Following overnight precipitation, the sample was centrifuged at 16500 ×g for 20 min at 4°C and the RNA pellet was washed twice with 70% ice-cold ethanol and finally with ice-cold absolute ethanol. The resultant RNA pellet was air-dried for 10 min and then resuspended with 30 μL to 50 μL of DEPC-treated water depending on pellet size.

### Removal of Genomic DNA (gDNA)

For the removal of gDNA, RNA sample was treated with RQ1 RNAse-Free DNAse (Promega) in a 1.5 mL microcentrifuge tube according to the manufacturer’s instructions. The 10 μL DNAse digestion reaction was set up with: 1 μL of RQ1 RNase-Free DNase 10X Reaction Buffer, 1μL of RQ1 RNase-Free DNase and 8 μL of RNA sample. Following an hour of incubation at 37°C, the solution was placed on ice and then topped up with to a volume of 100 μL DEPC-treated water. An equal amount of chloroform: isoamyl alcohol (24:1) was added, and the mixture was hand-shaken to mix prior to centrifugation at 16500 ×*g* for 5 min at 4°C. Following centrifugation, the supernatant was transferred to a new 1.5 mL tube and 1/10 volume of 8M LiCl_2_ was added, mixed well and incubated at −20°C overnight.

Following overnight precipitation, the sample was centrifuged at 16500 ×*g* for 15 min at 4°C and the pellet was washed twice with 70% ice-cold ethanol and finally with ice-cold absolute ethanol. The resultant pellet was air-dried and then dissolved with 10 μL to 15 μL of DEPC-treated water. The dissolved sample was then stored in −80°C freezer until further use.

### RNA Analyses

The quality of the extracted RNA sample was checked by agarose gel electrophoresis (1% agarose gel), measurement of absorbance ratios at 230 nm, 260 nm and 280 nm using NanoDrop spectrophotometer (Thermo Fisher Scientific, Delaware, USA). RNA integrity was determined using the Agilent 2100 Bioanalyzer (Agilent Technologies, California, USA). Concentration of RNA was quantified using a Qubit fluorometer (Life Technologies, California, USA).

## RESULTS

### Gel Electrophoresis of Extracted RNA

**Figure 1:**
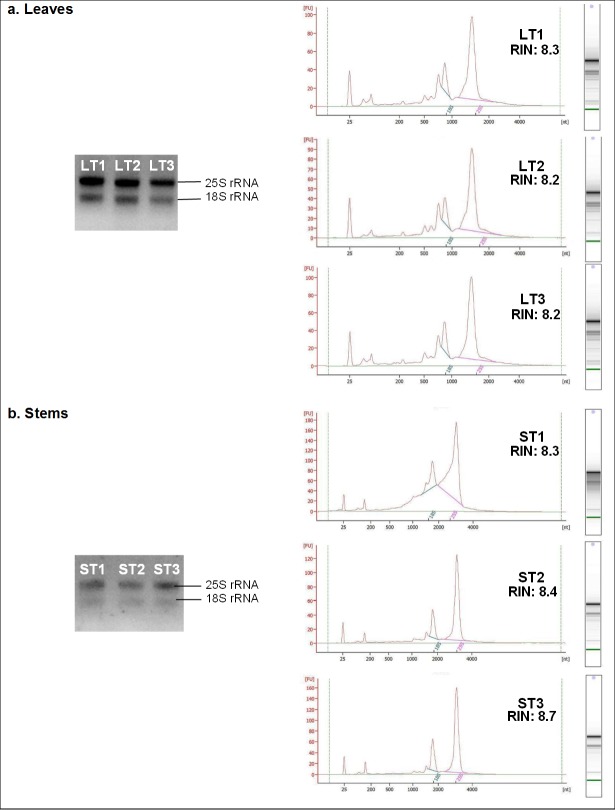
Agarose gel electrophoresis and the respective electropherograms of the total RNA samples extracted from (a) leaves, and (b) stems, of *Stevia rebaudiana*. Two distinct rRNA bands and peaks were clearly observed, for each sample, in both the gel and electropherograms respectively, signifying high RNA quality and minimal degradation. LT1-LT3: Replicates of RNA extracts from leaf tissues (400–600 ng); ST1-ST3: Replicates of RNA extracts from stem tissues (300–400 ng).

The quality of extracted RNA samples was first examined via gel electrophoresis (Fig. 1). Based on the gel pictures, two distinct rRNA bands can be observed clearly, with the higher band of 25S unit rRNA brighter than the lower band of 18S unit rRNA.

### Quantity, Purity and Integrity Analyses of Extracted RNA

RNA samples were isolated from three replicates of each of the leaf and stem tissues. In order to assess sample purity and quality with high accuracy, the absorbance ratios of 260/280 and 260/230 of total RNA were analysed in combination with overall spectral quality using a NanoDrop spectrophotometer (Table 1). All the RNA samples had 260/280 absorbance ratios of more than 2.0 and less than 2.2, suggesting that the samples had little or very minimal protein contamination. The 260/230 absorbance ratios obtained for all the samples were higher than 1.95, reflecting minimal or low contamination of polysaccharides and polyphenols in the RNA samples. The amounts of RNA samples yielded from 100 mg of leaves (stems) were more than 4000 ng.

RNA integrity was then evaluated using the Agilent 2100 Bioanalyzer. All the RNA samples extracted from both leaves and stems had RIN values of more than 8 (as shown in Fig. 1 and listed in Table 1), representing high integrity and quality of RNA.

## DISCUSSIONS

### CTAB Extraction Protocol

The CTAB protocol modified in this study was based on a reported protocol by Chang *et al*. (2016) on a gymnosperm plant, *Platycladus orientalis*. In their study, the RNA sample yielded was of extremely low purity levels (the 260/280 absorbance ratio was 1.05 and the 260/230 absorbance ratio was 0.79). In our study, we modified their protocol as follows: (1) the addition of PVP to the CTAB buffer, (2) higher centrifugation speed used in this study (16500 ×*g*), (3) inclusion of the usage of 100% ethanol at the end of washing step, and (4) an additional gDNA removal step. These modifications yielded high quality RNA samples in our study (with RIN > 8 and both absorbance ratios > 2).

CTAB is a detergent that solubilises plant cell wall and lipid membranes of internal organelles, and also denatures proteins to release the cell contents (Sánchez *et al*. 2016). The addition of both PVP and β-mercaptoethanol into the CTAB buffer had been proven successful to remove polysaccharides and phenolic compounds (Gao *et al*. 2016) present in different plant tissues of black and white mangroves (Rubio-Piña & Zapata-Pérez 2011), peach (Tong *et al*. 2012), Maqui berry (Sánchez *et al*. 2016), moringa tree (Gao *et al*. 2016), and pericarps of mangosteen (Abdul-Rahman *et al*. 2017). PVP functions to avoid the release of phenolic substances when crushing plant materials by binding to the polyphenol compounds by its CO-N= group and it also prevents browning effect due to presence of polyphenols (Wang & Vodkin 1994; Chang *et al*. 2016). β-mercaptoethanol was added to the extraction buffer as a reducing agent to prevent oxidation, and to inhibit the activity of RNAse by altering the native conformation of the enzyme for normal functionality (Rubio-Piña & Zapata-Pérez 2011; Sánchez *et al*. 2016). LiCl_2_ precipitation was carried out since LiCl_2_ precipitates RNA readily at certain concentrations but not DNA and protein (Chang *et al*. 2016; Sánchez *et al*. 2016). LiCl_2_ acts as a strong dehydrant that is able to decrease the solubility of RNA and thus RNA is pelleted and the remaining polysaccharides and unoxidised polyphenols are remained in the supernatant and removed (Green & Sambrook 2012). The RNA pellet obtained after overnight precipitation and centrifugation was washed twice with 70% ethanol to remove excess LiCl_2_ present in the tube and finally with 100% ethanol to ease the drying of the clean RNA pellet contained in the tube.

We have included an additional DNA removal step that is absent in the protocol of Chang *et al*. (2016) to completely remove trace amount of gDNA that might be present in the extracted samples. The gDNA removal step illustrated in this study, coupled with the chloroform: isoamyl alcohol (24:1) extraction and short centrifugation steps, was found to be highly effective to further remove excessive salt and contaminants that were carried over during RNA isolation, shown by the absence of gDNA band on the agarose gel (Fig. 1) and the high RNA Integrity Numbers or RINs (Table 1) obtained.

### Comparison with Previously-Reported RNA Extraction Methods from *Stevia rebaudiana*

Commercially-available kits, such as Qiagen RNA plant mini kit (Qiagen, Hilden, Germany) and Spectrum Plant Total RNA Kit (Sigma-Aldrich, Missouri, USA), had been successfully used to isolate RNA from the leaf tissues of *Stevia rebaudiana* (J Chen *et al*. 2014; Kim *et al*. 2015). The RIN numbers of the RNA samples were fairly high (RIN > 7), allowing these samples to be used for library preparation and RNA-Seq. Although commercial kits are known to isolate RNA quickly and with high quality, the yield is usually lower (Chang *et al*. 2016; Gao *et al*. 2016) and the cost incurred for such extraction is higher compared to conventional method.

Thus, conventional methods such as the acid guanidinium thiocyanate-phenol-chloroform extraction method developed by Chomczynski and Sacchi (1987), and the phenol/SDS based IHBT protocol developed by Ghawana *et al*. (2011) were also utilised in several studies to isolate RNA from stevia species (Madhav *et al*. 2013; Modi *et al*. 2014; Michalec-Warzecha *et al*. 2016). The RNA samples extracted using these conventional methods were successfully used for downstream applications including RT-PCR and qPCR despite the absence of the reported RNA quality in their studies. However, no single published protocol reported yet for high-quality and high-yield RNA extraction from *Stevia rebaudiana* with CTAB as basis.

The integrity of RNA sample isolated in our study was validated via agarose gel electrophoresis and subsequently assessed using Agilent 2100 Bioanalyzer. The bioanalyzer takes into account the entire electrophoretic trace of RNA samples and RNA qualities were classified into RINs of 1 (most degraded profile) to 10 (most intact profile) (Mueller *et al*. 2016). Thus far, there is no fixed range of RINs that must be adhered to decide the suitability of the RNA sample for sensitive downstream applications such as RNA-Seq. Ideally, RNA with RIN value more than 7 is preferable for sensitive application such as RNA-Seq (Sigurgeirsson *et al*. 2014), but RNA with RIN as low as 5.4 (Sánchez *et al*. 2016) had also been used for RNA-Seq of plant tissue samples. This is because plants have a variety of rRNA sizes due to the presence of plastid ribosomes, thus generating a complexity in RIN reading, as the RIN algorithm was originally developed to evaluate the integrity of RNA from mammalian tissues (Johnson *et al*. 2012). However, it is desirable to use samples with RIN > 7 as previous studies demonstrated that sequencing results and gene expression profiles are influenced by RIN value (E A Chen *et al*. 2014; Kukurba & Montgomery 2015; Reiman *et al*. 2017). In the studies conducted on *Stevia rebaudiana* by J Chen *et al*. (2014) and Kim *et al*. (2015), both groups only used RNA samples with RIN > 7 for RNA sequencing. The results from our study are very promising where RNAs of high yield with RIN > 8 were successfully obtained from the leaf and stem tissues. Thus, the protocol developed in this study can be an affordable alternative method to extract RNA of high quality from *Stevia rebaudiana* effectively.

## CONCLUSION

The modified CTAB-based extraction protocol used in this study was effective and reliable in isolating RNA samples from leaves and stems of *Stevia rebaudiana* with high integrity, quality and yield (with RIN values more than 8) that are suitable for subsequent molecular applications such as RNA sequencing.

## Figures and Tables

**Table 1:** Total amount, RIN number and absorbance ratios of respective RNA samples extracted from the (A) leaves, and (B) stems, of *Stevia rebaudiana*.

	Total RNA amount (ng)	RIN Number	Absorbance ratio	

			OD260/OD280	OD260/OD230
a. Leaves				
LT1	6035.7	8.3	2.07	2.24
LT2	4323.0	8.2	2.03	2.43
LT3	5518.8	8.2	2.11	2.11
b. Stems				
ST1	4796.7	8.3	2.04	2.57
ST2	4132.8	8.4	2.06	2.71
ST3	4536.0	8.7	2.11	1.95
